# Videofluoroscopic swallow study in dysphagia stroke patients

**DOI:** 10.3389/fneur.2026.1837979

**Published:** 2026-07-07

**Authors:** Martina Kelblová, Jiří Vaníček, Viktor Weiss, Vladimír Červeňák, Tomáš Křivka, Karin Ďurčanská

**Affiliations:** 1 Department of Medical Imaging, St. Anne’s University Hospital and Faculty of Medicine, Masaryk University, Brno, Czechia; 2 The First Department of Neurology, Comprehensive Stroke Center, St. Anne‘s University Hospital and Faculty of Medicine, Masaryk University, Brno, Czechia

**Keywords:** computed tomography, corticobulbar tract, dysphagia, magnetic resonance imaging, stroke, swallowing act, swallowing disorder, videofluoroscopic swallow study

## Abstract

**Background and objectives:**

Dysphagia is a common symptom of ischemic stroke. Severe complications include aspiration pneumonia, malnutrition, and dehydration. A videofluoroscopic swallow study (VFSS) allows for detailed dynamic and morphological examination of the swallowing act and therapy adjustment. Our study aimed to determine relationships between ischemic lesion locations and the character of swallowing disorder, as well as the frequency and severity of aspiration.

**Methods:**

This single-center retrospective study involved 27 post-stroke dysphagia patients, treated at our institution. The CT stroke examination protocol results of all patients were negative. They underwent brain magnetic resonance imaging (MRI), confirming the diagnosis of stroke and identifying the exact lesion location. Subsequently, all patients underwent VFSS with a modified barium swallow impairment profile (MBSImP) assessment, including calculating oral impairment (OI) and pharyngeal impairment (PI) variables. Aspiration was scored according to Rosenbek‘s Penetration-Aspiration Scale (PAS). We explored the relationships between OI, PI, and PAS variables and lesion locations.

**Results:**

Oral phase impairment (as represented by OI variables) differed significantly by lesion location, with greater impairment in supratentorial lesions (*p* < 0.001). Pharyngeal phase impairment showed no statistically significant differences by lesion location. Higher impairment in infratentorial lesion location was not statistically significant (*p* = 0.057). We found no corellation between supra- or infratentorial lesion location and the frequency or severity of aspiration.

**Conclusion:**

The location of ischemic stroke lesions in the corticobulbar tract can indicate the character of the swallowing disorder to some extent. The oral phase is statistically significantly more affected in supratentorial lesions. Damage to the corticobulbar tract is often associated with aspiration, with an unclear relationship to the lesion location. Including VFSS in the diagnostic algorithm for dysphagic patients with mild or moderate stroke appears beneficial regardless of the lesion location.

## 1 Introduction

Stroke is the third leading cause of death worldwide and the second leading cause of disability, according to WHO data (1). Most strokes, more than 80%, are ischemic. Multidisciplinary teams at stroke centers provide the comprehensive management of patients with acute ischemic stroke. Management includes diagnostics, therapy, and rehabilitation (2–4). Diagnosis is based on clinical and laboratory tests and imaging methods, primarily using computed tomography (CT) or magnetic resonance imaging (MRI) (3–5). Ischemic stroke is treated with reperfusion via intravenous thrombolysis and/or mechanical recanalization of the vascular occlusion, followed by secondary preventive measures (2–4). The goal of treatment is not only to reduce mortality but also to maintain the patient’s functional independence. This outcome is achieved through the timely initiation of reperfusion therapy and early rehabilitation. Swallowing disorder or dysphagia is a severe symptom of stroke that may cause aspiration pneumonia, malnutrition, and dehydration (6). In patients with dysphagia, rehabilitation procedures include speech-language therapy based on results of a clinical swallowing examination, which must be confirmed by instrumental examination, such as a videofluoroscopic swallow study (VFSS) or a flexible endoscopic evaluation of swallowing (FEES) (6–11).

The swallowing act is a complex physiological process that transports food mixed with saliva from the mouth to the stomach while protecting the airways from aspiration (10). Neural control primarily involves the trigeminal nerve (V), the facial nerve (VII), and the lateral mixed system (IX, X, XI) with centers in the brainstem. All of these are controlled by cortical centers, primarily in the precentral gyrus, via upper motor neurons of the corticobulbar tract (10, 12, 13). The swallowing act is conventionally divided into four anatomically and temporally distinct phases: the oral preparatory, oral transport, pharyngeal, and esophageal phases (10, 11). The oral preparatory phase involves holding a food bolus. It consists of the lip closure, forming a velolingual seal, and sealing the tongue margins against the upper jaw to prevent ventral leakage or drooling, dorsal leakage, and lateral buccal bolus escape. During the oral transport phase, the bolus is moved to the pharynx by gradually rolling the tongue from the tip to the base against the hard palate. The pharyngeal phase is the most complex phase of the swallowing act. It involves a physiological sequence of actions, including soft palate elevation, also known as the velopharyngeal closure to prevent the bolus from leaking into the nasal cavity, the elevation and anterior movement of the hyoid and larynx, laryngeal vestibular closure to prevent aspiration, and the opening of the upper esophageal sphincter. Finally, the esophageal phase involves the transport of food between the upper and lower esophageal sphincters via esophageal peristalsis (9–11, 14). Dysphagia may be associated with food entering the airways (penetration above the vocal cords and aspiration below them). It may cause aspiration pneumonia, especially so-called silent aspiration without coughing to expel the food (10). Accurate evaluation of the affected swallowing act phases, as well as aspiration when present, requires more than clinical observation alone.

Despite the widespread clinical use of speech-language pathologist (SLP) examinations in neurological stroke units for dysphagia assessment, their accuracy remains limited (6, 15, 16). Therefore, standardized instrumental examinations to validate these disorders are crucial. Two complementary standardized instrumental methods, VFSS, also known as modified barium swallow (MBS), and FEES are the most widely used. VFSS is a dynamic fluoroscopy examination that provides high temporal and spatial resolution of all phases of the swallowing act via high-frequency digital recording with a barium-based radiopaque medium. FEES, in turn, uses a flexible endoscope that, once introduced into the pharynx, provides a visualization of the end of the oral transport phase and the start of the pharyngeal phase of swallowing. Each method has advantages: VFSS captures the entire swallowing act and detects aspiration more precisely (with the patient in a standing or sitting position within the radiographic system), whereas FEES is an easy-to-perform bedside technique, even in immobile patients (6–9, 17). The choice of an appropriate method depends on the patient’s clinical scenario. VFSS is reserved for patients with milder forms of stroke with National Institutes of Health Stroke Scale (NIHSS) scores in the range of 0 to 5 or 6 to 10, and, exceptionally, 11 to 15. FEES is feasible even in patients with more severe forms of stroke, with NIHSS scores of 16 to 20 or 21 to 25 (6).

Nowadays, FEES is widely available in many stroke centers (6). While VFSS, which requires transporting the patient to the radiology department and specialized, trained staff, has varying availability at stroke centers, with greater access in countries like the USA and Canada compared to others (17). We considered whether patients with specific ischemic lesion locations should be prioritized for VFSS.

Our study aimed to determine whether the supratentorial or infratentorial location of ischemic stroke lesions can indicate the character of swallowing disorder detected by VFSS and whether the location is associated with the risk and severity of aspiration.

## 2 Materials and methods

### 2.1 Study design

This single-center retrospective study included 27 first-ever stroke patients with dysphagia examined within our hospital’s cerebrovascular program, treated between February 2021 and October 2025. Upon arrival, the patients were examined by expert neurologists, who assessed their neurological deficits and classified them according to the NIHSS before proceeding with further clinical procedures. An SLP expert evaluated the swallowing condition and recommended VFSS depending on the case. Patients with milder clinical scenarios who were classified as having had a minor stroke (0–5 points), mild stroke (6–10 points), or moderate stroke (11–15 points), underwent VFSS for accurate dysphagia assessment and were included in our study. Patients who had severe strokes with dysphagia, evaluated via FEES, were excluded. All patients underwent a standard brain computed tomography (CT) according to the CT stroke protocol and early brain MRI to confirm the diagnosis of stroke and determine the exact lesion location, followed by VFSS with swallowing profile assessment, and aspiration scoring. These examinations were evaluated by expert radiologists who were blinded to the patients’ personal and clinical data.

Exclusion criteria were severe forms of stroke, a history or imaging finding of having had a previous stroke, and other causes of neurological deficit other than ischemia, such as a hemorrhage, tumor, seizure, infection, and so forth.

### 2.2 Computed tomography (CT)

Patients underwent a standard brain CT examination on a Philips iCT 256 scanner according to a CT stroke protocol that included a native CT, CT angiography (CTA), and CT perfusion imaging (CTP) (4, 5).

Native CT scans were performed in all patients suspected of stroke to rule out other pathologies such as bleeding or tumors, and to detect early ischemic changes and assess the extent of the ischemia. The standardized 10-point, Alberta Stroke Program Early CT Score (ASPECTS) system was used to assess early CT ischemic changes in middle cerebral artery (MCA) strokes (5, 18).

After bleeding or other causative pathologies were ruled out, patients suspected of acute stroke underwent multiple-phase (three-phase) CTA. The first phase covered the range from the aortic arch to the vertex, evaluating the entire cerebral vasculature intra- and extracranially, identifying the occlusion, and assessing the access route if a neurointerventional recanalization was scheduled. The next two phases were performed from the skull base to the vertex to evaluate collateral circulation (5, 18, 19).

Finally, the protocol included CTP, a dynamic post-contrast examination to characterize cerebral perfusion. Perfusion parameters are evaluated to identify the ischemic core (representing irreversibly damaged tissue) and the ischemic penumbra (representing salvageable hypoperfused brain tissue) (5, 20–22).

CT examinations were acquired with the following parameters: native CT (slice thickness 0.90 mm, spacing 0.45 mm, 120 kV, 251 mAs), CTA (slice thickness 0.90 mm, spacing 0.45 mm, 100 kV, 220 mAs, contrast agent Iomeron 400 at a dose of 60 mL injected consecutively at 4 and 5 mL/s), and CTP (slice thickness 10.00 mm, spacing 10.00 mm, 80 kV, 124 mAs, contrast agent Iomeron 400 at a dose of 40 mL injected at 5 mL/s, temporal resolution 0.50 s, total acquisition time 4 s, coverage 80 mm). The images were analyzed by two independent expert radiologists (TK and VČ), with 10 years of experience each, consecutively using two automatic software tools (RAPID, Brainomix). In the event of a discrepancy, a third expert radiologist (KĎ) also assessed the findings.

### 2.3 Magnetic resonance imaging (MRI)

All patients with negative CT stroke protocol results underwent a native MRI examination for suspected stroke on a Philips Ingenia 1.5 T scanner. The protocol included diffusion-weighted imaging (DWI) for the accurate detection of acute ischemic changes, fluid-attenuated inversion recovery (FLAIR) for characterization of acute or subacute ischemic lesions, and gradient-echo (GRE) sequences for exclusion or visualization of hemorrhages (5, 23, 24).

MRI sequences were acquired with the following parameters: DWI (TR 6000.0 ms, TE 105.1 ms, b-values 0 and 1,000 s/mm^2^, slice thickness 4 mm), FLAIR (TR 8400.0 ms, TE 126.1 ms, TI 2216.7 ms, slice thickness 4 mm), and GRE (TR 940.0 ms, TE 25.0 ms, flip angle 20°, slice thickness 4 mm). The images were assessed by two independent expert radiologists (TK and VČ), with 10 years of experience each. In the event of a discrepancy, a third expert radiologist (KĎ) also assessed the findings.

### 2.4 Videofluoroscopic swallow study (VFSS)

Another expert radiologist (MK), trained in VFSS examination and MBSImP scoring, and a speech-language pathologist (SLP) performed the early VFSS procedure within three to 5 days after admission to the stroke center according to the guidelines (7, 8, 10), using the interventional radiographic system Siemens Artis Zee MP with a fluoroscopic recording frequency of 30 frames per second.

The experts followed the Logemann standard in a lateral view, capturing the oral and nasal cavities, pharynx, larynx, and proximal esophagus. Each patient was gradually given small and larger sips of a barium contrast agent with the consistency of a thin liquid (dilution contrast with water 1:1), followed by continuous drinking, then a sip of thickened liquid (undiluted contrast agent), and finally a puree (a mixture of contrast agent and a medical thickener) and solid food (a baby biscuit coated in the contrast agent). The order and amount of consistencies were set based on the results of the SLP assessment, starting with those with the least difficulties and the lowest risk of aspiration and progressing to those with higher risk. In case of significant aspiration, the procedure was terminated. Finally, the patient was examined in an anteroposterior view to detect lateral asymmetries, such as unilateral pharyngeal residues, and also to assess esophageal clearance. A more detailed examination of the esophageal phase is not a standard part of VFSS because it is not accessible to therapeutic exercise maneuvers (6, 8, 10).

Standardized scoring systems based on VFSS results have been developed, such as the MBSImP (Modified Barium Swallow Impairment Profile), which was published and approved by the American Speech-Language-Hearing Association (ASHA) (25). MBSImP contains 17 physiological components of the swallowing act, including oral, pharyngeal, and minimal esophageal components. Fourteen physiological components of the MBSImP scale were evaluated in our cohort.

To assess the degree and severity of contrast agent permeation into the airways, Rosenbek’s Penetration-Aspiration Scale (PAS) was used: A score of 1 is a normal finding, whereas, scores of 2 to 5 indicate penetration and, scores of 6 to 8, aspiration. A score of 8 indicates „silent aspiration “without coughing (7, 26).

Both the MBSImP and PAS scores were re-evaluated by an expert radiologist (MK) for all patients at a 3-month interval for subsequent intra-rater reliability assessment.

### 2.5 Statistical analysis

Data were tested for normality using the Shapiro–Wilk test and graphically using Q-Q plots and histograms. Continuous variables are presented as median (interquartile range). Categorical variables are presented as numbers with percentages. Group comparisons for continuous variables were performed using the Mann–Whitney U test. Pearson‘s Chi-Square test was used to assess the relationship between two categorical variables. A two-sided *p*-value of 0.05 was considered statistically significant. Statistical analysis was performed in the programming language R (version 4.2.1) using the integrated development environment RStudio (release 2025.09.02, RStudio, PBC). Weighted Cohen‘s kappa was used to assess intra-rater reliability for MBSImP and PAS scoring.

## 3 Results

### 3.1 Study group

Twenty-seven first-ever stroke patients with dysphagia (20 male, 7 female) were included in the study. Demographic and clinical data are shown in Table 1.

**Table 1:** Demographic and clinical characteristics of the study cohort stratified by lesion location.

Variable	Total (*N* = 27)	Supratentorial (*N* = 19)	Infratentorial (*N* = 8)
Age (years)	72 (64, 83)	69 (60, 83)	76 (74, 81)
Sex (*N*(%))			
Male	20 (74)	13 (68)	7 (87)
Female	7 (25)	6 (31)	1 (12)
NIHSS	3 (2, 6)	4 (3, 6)	3 (1, 5)

NIHSS, National Institutes of Health Stroke Scale.

### 3.2 Brain imaging

The results of the CT stroke protocol in all our retrospectively analyzed patients were negative, that is, they showed no early native CT stroke signs with ASPECTS 10, no cerebral vessel occlusions on CTA scans, and no ischemic core and penumbra according to CTP analysis. Hemorrhages, tumors, or other causative pathologies visible on CT scans were also excluded.

**Figure 1:**
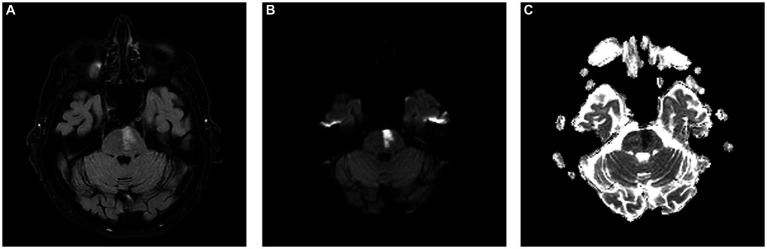
Brain MRI. Acute left pontine ischemia. **(A)** FLAIR transverse image, **(B)** DWI transverse image, **(C)** ADC transverse image.

**Figure 2:**
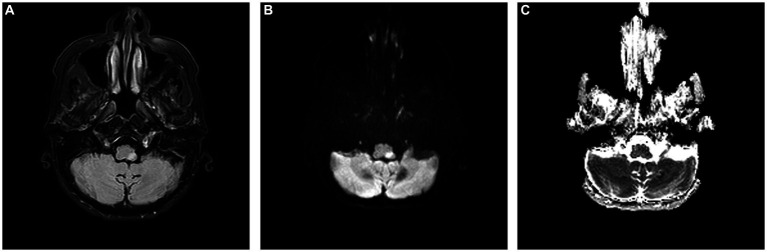
Brain MRI. Acute ischemia of the left medulla oblongata. **(A)** FLAIR transverse image, **(B)** DWI transverse image, **(C)** ADC transverse image.

**Figure 3:**
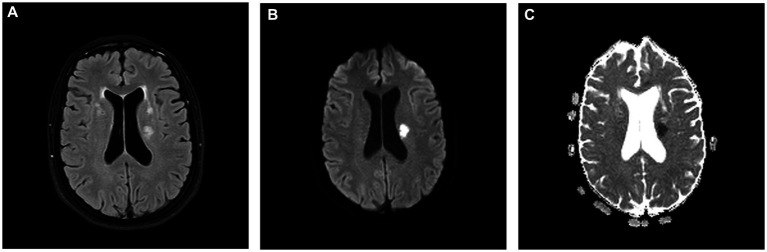
Brain MRI. Acute ischemia of the left corona radiata. **(A)** FLAIR transverse image, **(B)** DWI transverse image, **(C)** ADC transverse image.

**Figure 4:**
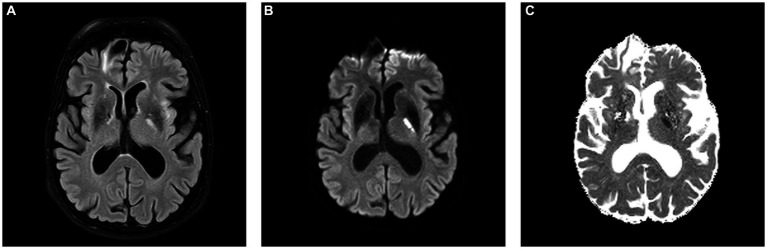
Brain MRI. Acute ischemia of the posterior limb of the left internal capsule. **(A)** FLAIR transverse image, **(B)** DWI transverse image, **(C)** ADC transverse image.

**Figure 5:**
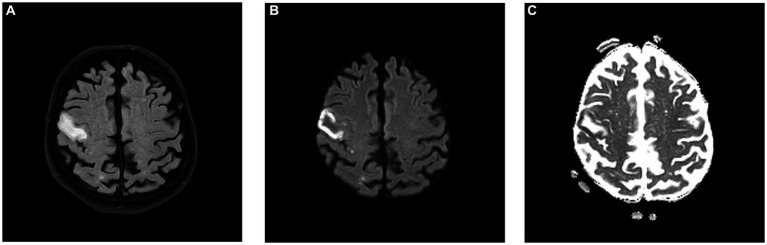
Brain MRI. Acute ischemia of the right precentral gyrus. **(A)** FLAIR transverse image, **(B)** DWI transverse image, **(C)** ADC transverse image.

Brain MRIs revealed infratentorial ischemic lesions in eight patients and supratentorial ischemic lesions in 19 patients (Figures 1–5).

### 3.3 Swallowing study

**Figure 6:**
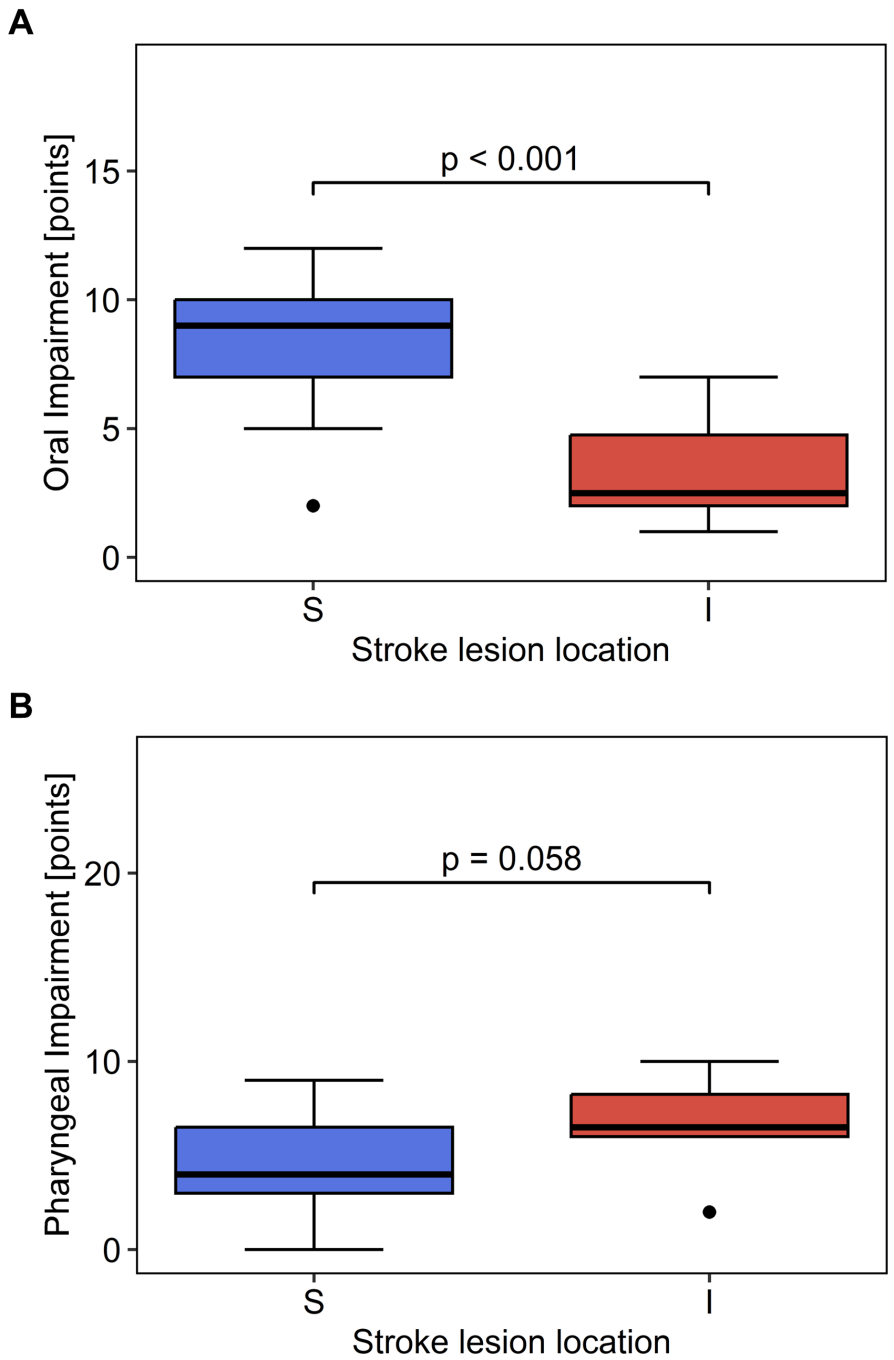
Boxplots of the oral **(A)** and pharyngeal **(B)** impairment by stroke lesion location (S - supratentorial, I - infratentorial).

OI (oral impairment) and PI (pharyngeal impairment) are represented as both point scores (0–19 for OI, 0–26 for PI) and percentage scores. OI differed significantly between supratentorial and infratentorial lesion groups (Table 2) (Figure 6).

**Table 2:** Oral and pharyngeal impairment by lesion location.

Variable	Supratentorial (*N* = 19)	Infratentorial (*N* = 8)	*p*-value
OI (points)	9.0 (7.0, 11.0)	2.5 (2.0, 5.5)	<0.001
OI (%)	47.4 (36.8, 57.9)	13.2 (10.5, 29.0)	
PI (points)	4.0 (3.0, 7.0)	6.5 (6.0, 8.5)	0.058
PI (%)	15.4 (11.5, 26.9)	25.0 (23.1, 32.7)	

OI, Oral impairment; PI, Pharyngeal impairment.

**Figure 7:**
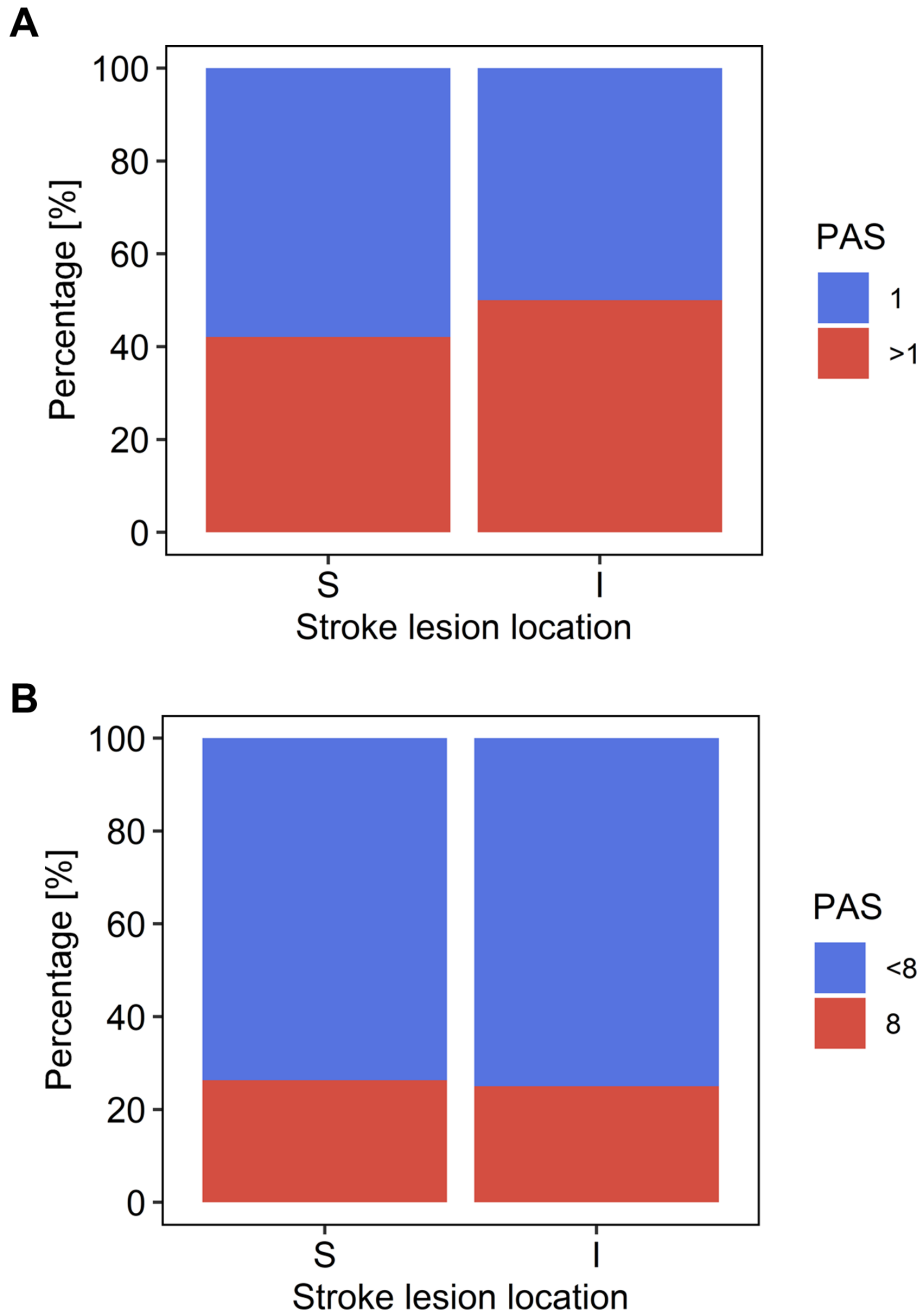
Stacked box plots of Penetration-Aspiration Scale (PAS) by stroke lesion location (S - supratentorial, I - infratentorial). **(A)** Proportion of PAS ˃ 1 (any penetration or aspiration) versus PAS 1 (no peneration/aspiration). **(B)** Proportion of PAS 8 (silent aspiration) versus PAS < 8. Colours indicate PAS category, and the height of each segment corresponds to its percentage of the total in each group.

PAS scores indicated that 15 patients (55.6%) had no penetration or aspiration (PAS 1), while 12 patients (44.4%) had evidence of penetration or aspiration (PAS ˃ 1). Among these, seven patients (25.9%) exhibited silent aspiration (PAS score of 8). Stratified by lesion location, the proportion of patients with any aspiration was 42.1% in supratentorial lesions and 50.0% in infratentorial lesions, whereas silent aspiration occurred in 26.4 and 25.0% of patients, respectively (Table 3; Figure 7). Overall, PAS scores were similar between the lesion groups.

**Table 3:** Penetration-Aspiration scores distributed by lesion location.

PAS	Total (*N* = 27)	Supratentorial (*N* = 19)	Infratentorial (*N* = 8)	*p*-value
1	15 (55.6%)	11 (57.9%)	4 (50.0%)	-
2	1 (3.7%)	1 (5.3%)	0 (0.0%)	
3	1 (3.7%)	1 (5.3%)	0 (0.0%)	
4	1 (3.7%)	0 (0.0%)	1 (12.5%)	
5	0 (0.0%)	0 (0.0%)	0 (0.0%)	
6	1 (3.7%)	0 (0.0%)	1 (12.5%)	
7	1 (3.7%)	1 (5.3%)	0 (0.0%)	
8	7 (25.9%)	5 (26.3%)	2 (25.0%)	
PAS>1 vs. PAS 1	12 (44.4%)	8 (42.1%)	4 (50.0%)	> 0.999
PAS 8 vs. PAS 1–7	7 (25.9%)	5 (26.4%)	2 (25.0%)	> 0.999

PAS, Penetration-Aspiration Scale.

Intra-rater reliability of MK, assessed using Cohen’s quadratic kappa for MBSImP and PAS scoring, showed the following kappa values: OI: 0.91 [95% CI (0.82;1.00)], PI: 0.87 [95% CI (0.75; 0.99)], PAS: 0.96 [95% CI (0.92; 1.00)].

**Figure 8:**
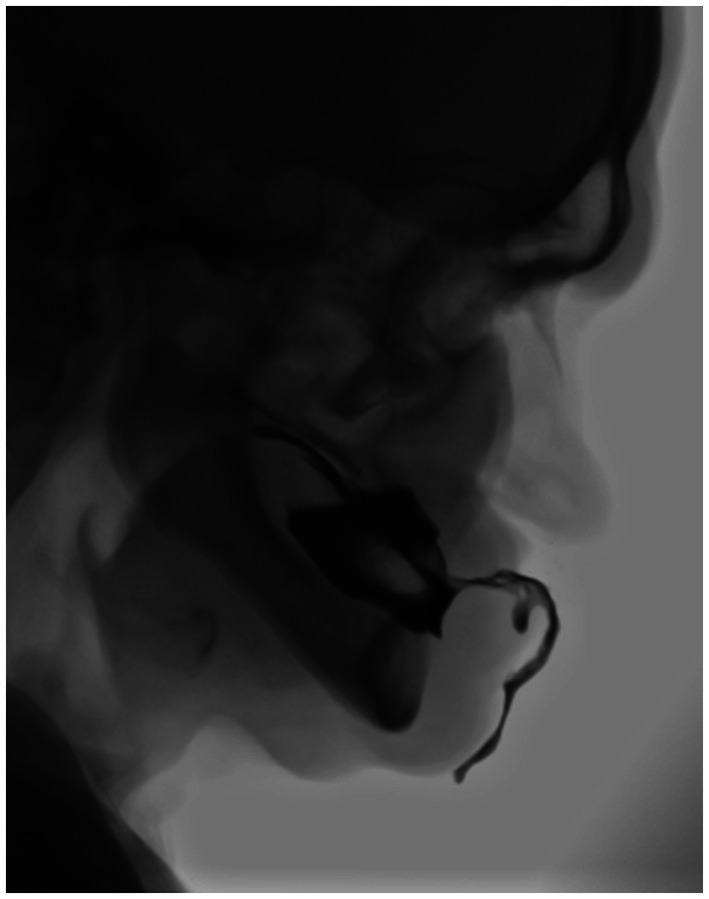
VFSS, lateral view. Oral preparatory phase; lip closure impairment with ventral leak beyond mid-chin (drooling); tongue control impairment with lateral escape to buccal cavity.

**Figure 9:**
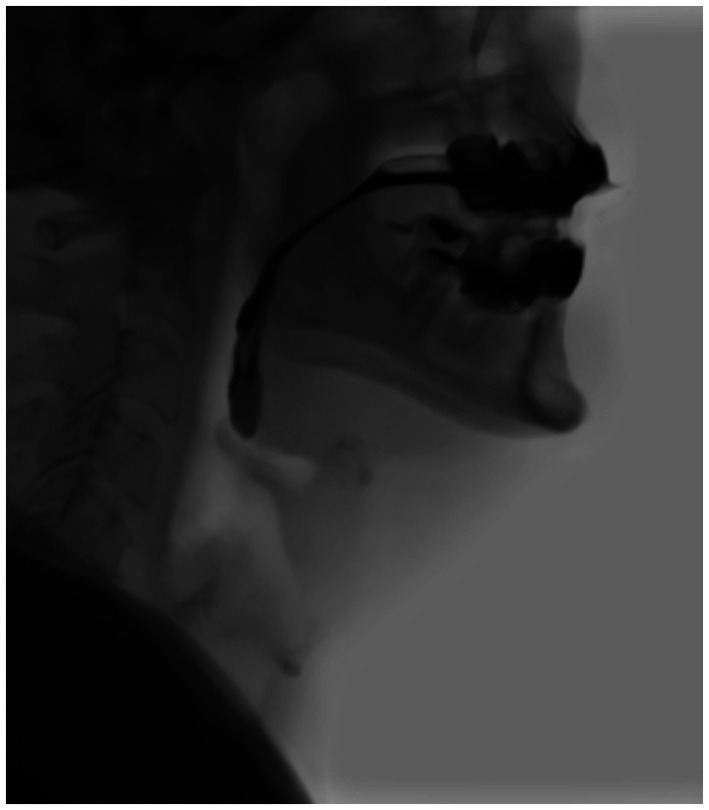
VFSS, lateral view. Oral preparatory phase; tongue bolus control impairment with dorsal leak of more than half of the bolus.

**Figure 10:**
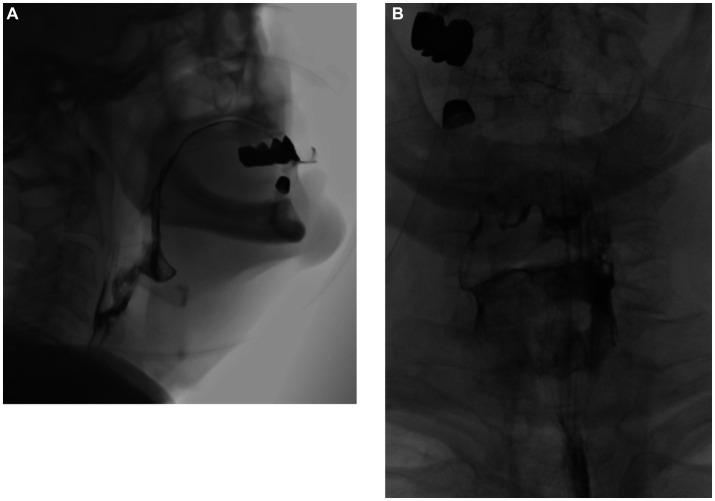
VFSS. **(A)** lateral view **(B)** anteroposterior view. Pharyngeal phase impairment with residues in the left valleculae and piriform sinuses. Acute ischemia of the left medulla oblongata.

**Figure 11:**
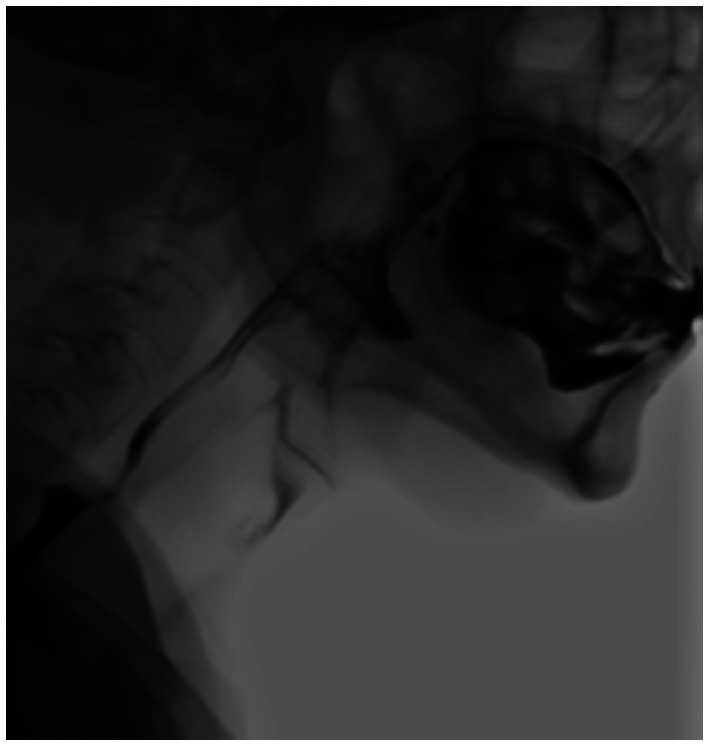
VFSS, lateral view. Aspiration—contrast agent permeation beneath the vocal cords. Simultaneously, lateral bucal escape of the bolus occurs.

**Figure 12:**
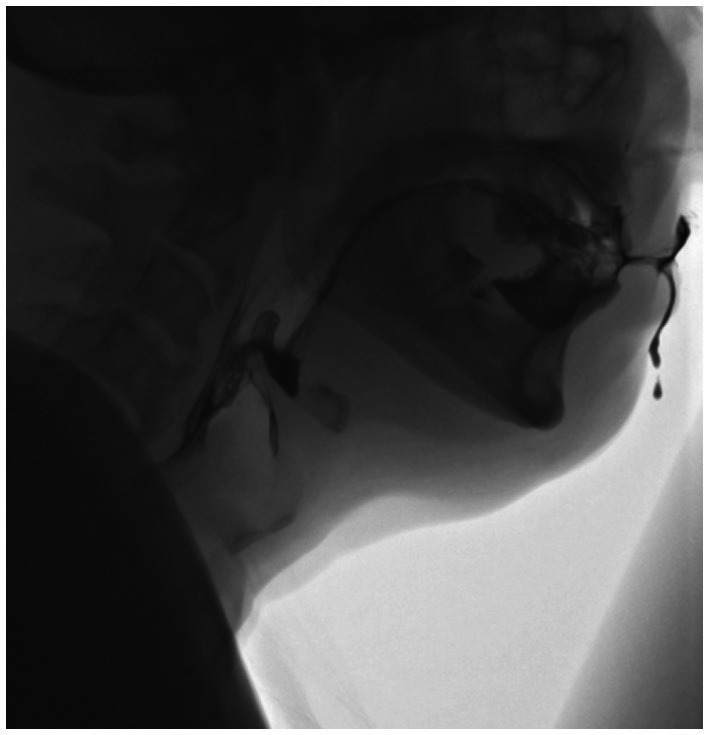
VFSS, lateral view. Penetration—contrast agent permeation above the vocal cords. Simultaneously, drooling is depicted.

The pathological VFSS findings of our patients are demonstrated in Figures 8–12.

## 4 Discussion

We made three main findings. First, the location of an acute ischemic lesion in patients with dysphagia can indicate the character of the swallowing disorder to some extent, with greater impairment of the oral domain in supratentorial lesions. Second, it is appropriate to evaluate not the entire oral and pharyngeal domains, but their individual components, a task facilitated by the detailed, standardized MBSImP scale. Third, we also observed an association between ischemic stroke lesions along the cortico-bulbar tract and a relatively frequent occurrence of aspiration or penetration, which did not significantly differ depending on their supra or infratentorial location.

Other researchers have reported on differences in oral and pharyngeal phase impairment depending on lesion location in patients with dysphagia after stroke (27, 28). Kim et al. (27) showed that oral phase duration was significantly prolonged in supratentorial lesions, a finding similar to ours. Likewise, Yang et al. (28) reported greater pharyngeal phase impairment in brainstem lesions than in left-hemispheric lesions. We also found greater impairment of the pharyngeal phase in infratentorial lesions, although this finding did not reach statistical significance. However, this result of our study may be influenced by our smaller sample size, particularly in the infratentorial lesion group, which may increase the risk of a type II error.

The swallowing act is a complex, intricately regulated process in which the individual components of both the oral and pharyngeal domains are under the specific control of upper and/or lower motor neurons. Wilmskoetter et al. demonstrated a significant correlation between specific supratentorial ischemic lesion locations and individual pharyngeal components of swallowing (29). In our study, we also evaluated both the global and individual components and observed that the assessment depended on the lesion location. However, we have used a relatively broad classification of the lesion location due to the smaller sample size. We observed that evaluating individual components is crucial for speech-language pathologists to set up appropriate rehabilitation measures tailored to the patient.

According to our research findings, penetration and aspiration are relatively frequent in stroke patients with dysphagia. We did not prove a statistically significant association between penetration or aspiration frequency and severity and the lesion location. However, Daniels et al. demonstrated significantly higher PAS scores in infratentorial lesions (30).

Taken together, our findings suggest that the lesion location in stroke patients with dysphagia indicates the character of the swallowing disorder to some extent. However, the swallowing act is a complex process, with specific neural control of its individual components, and further research will be needed to achieve more detailed mapping. VFSS allows for a detailed examination of all swallowing act phases, enabling establishement of appropriate therapeutic and rehabilitation measures for the patient, thereby significantly reducing the risk of severe complications such as pneumonia, malnutrition, and dehydration.

Our study has several limitations. Our sample size was relatively small, particularly in the infratentorial lesion group, which may limit the generalizability of some of our results, in particular, those related to pharyngeal impairment and aspiration severity. The smaller size in our cohort was due to the fact that our comprehensive stroke center cares for many severe stroke patients who cannot undergo VFSS and are primarily referred for FEES. Likewise, because it was a retrospective, single-center study, we cannot rule out selection bias. In addition, inter-rater reliability between the radiologist and the SLP for MBSImP and PAS scoring could not be assessed due to the single-rater VFSS design. Furthermore, a significant portion of our sample was composed of older adults, who may have had presbyphagia. Finally, our inclusion period was moderately long due to the retrospective study design.

## 5 Conclusion

Ischemic stroke lesion location in patients with dysphagia can, to some extent, indicate the character of the swallowing disorder, such as greater oral phase impairment in supratentorial lesions. However, the complexity of neural control of the swallowing act requires further research with more detailed mapping. Corticobulbar tract damage is often associated with aspiration, with an unclear relationship to the lesion location. Including VFSS, which enables a precise evaluation of the entire swallowing act, in the examination algorithm appears beneficial in dysphagic patients with mild or moderate forms of ischemic stroke regardless of the lesion location.

## Data Availability

The raw data supporting the conclusions of this article will be made available by the authors, without undue reservation.
